# Inflammatory Biomarkers are Associated with Intimal Intracranial Artery Calcification

**DOI:** 10.7150/ijms.124210

**Published:** 2026-01-01

**Authors:** Dren Boshnjaku, Igor Petrov, Pranvera Ibrahimi, Edmond Komoni, Driton Shabani, Fisnik Jashari

**Affiliations:** 1 Medical Faculty Skopje 'Ss Cyril and Methodius/Skopje/North Macedonia.; 2 Clinic of Neurology/University Clinical Center of Kosovo/Pristina/Kosovo.; 3 Faculty of Medicine/University of Pristina “Hasan Pristina”/Prishtina/Kosovo.; 4 Department of Public Health and Clinical Science/Umeå University/Umeå/Sweden.; 5 Clinic of Cardiology/ University Clinical Center of Kosovo/Pristina/Kosovo.

**Keywords:** ischemic stroke, medial calcification, intimal calcification, red cell distribution width, red blood cell to platelet ratio

## Abstract

**Aim and Background**: The aim of this study was to evaluate the association between inflammatory markers, Red Blood Cell to Platelet Ratio (RPR) and the Red Cell Distribution Width (RDW%) with intracranial artery calcification subtypes and the severity of ischemic stroke.

**Methods**: A total of 118 patients with a mean age of 67.3±12.5 years, of whom 49% were female, with ischemic stroke were prospectively enrolled. The study was conducted at a single center. Intracranial artery calcification was evaluated using a standardized methodology, differentiating mainly intimal from mainly medial, mixed type (medial and intimal calcification), and lack of arterial calcification. In addition, a new categorical variable representing stroke outcome was created based on the change in modified Rankin Scale (mRS) scores between admission and discharge. Inflammatory markers were assessed based on CBC results collected from each patient with ischemic stroke.

**Results**: Patients with a predominantly intimal type of intracranial artery calcification had significantly higher RPR levels compared to those with predominantly medial or mixed-type calcification. In a multinomial logistic regression analysis adjusted for age, sex, diabetes, dyslipidemia, hypertension, smoking status, NIHSS at admission, and mRS at discharge, each standard deviation (SD) increase in RPR was associated with a higher likelihood of having intimal versus medial calcification (OR = 1.53; 95% CI: 1.06-2.11; p = 0.022), and intimal versus mixed-type calcification (OR = 1.34; 95% CI: 1.03-1.75; p = 0.027). Moreover, higher RPR levels were independently associated with worse functional outcomes after stroke (OR = 1.43; 95% CI: 1.09-1.88; p = 0.009).

**Conclusion**: We found that higher RPR were significantly correlated with intracranial intimal artery calcification and worse functional outcomes in patients with ischemic stroke, as indicated by increased modified mRS scores at discharge.

## Introduction

Ischemic stroke, the leading cause of disability globally, accounts for over 75% of all stroke cases 1. Inflammation plays a crucial role in the development and progression of ischemic stroke. Several simple blood biomarkers, including RDW%, RPR, platelet count, and Mean Platelet Volume (MPV), have been shown to reflect inflammatory activity and are associated with outcomes in various diseases, including cerebrovascular diseases 2, 3.

Intracranial artery calcification is another factor that influences stroke severity. Two distinct types of calcification are commonly observed: intimal and medial. These subtypes differ in their biological mechanisms and clinical effects.

Intimal calcification is linked to atherosclerosis and is associated with endothelial dysfunction, luminal narrowing, and the risk of plaque rupture. This can lead to thrombosis or distal embolism 4.

Medial calcification, on the other hand, occurs in the arterial media and is linked to arterial stiffness, elevated pulse pressure, and decreased blood flow to distant areas 5.

Inflammation directly impacts key processes in stroke, including ischemia, penumbra evolution, and infarct enlargement. It contributes to neuronal injury and clinical deterioration, partly by increasing hypercoagulability and oxidative stress 6, 7. This relationship suggests that inflammatory biomarkers such as RDW% and RPR may help assess both inflammation and stroke severity in clinical practice 8.

Understanding how inflammation interacts with intracranial calcification may also provide insights into different patterns of vascular disease. This perspective aligns with the growing recognition that inflammation, not only dyslipidemia, plays a central role in atherosclerosis and may guide future development of targeted anti-inflammatory therapies 9.

The objective of this study is to evaluate the association between inflammatory biomarkers, particularly RDW% and RPR, and the two subtypes of intracranial artery calcification. Additionally, the study aims to determine their impact on ischemic stroke severity and prognosis.

## Methods

### Patient data

In this prospective, single-center cohort study, we included 118 patients with acute ischemic stroke at the Clinic of Neurology, University Clinical Center of Kosovo, for a period of six months in 2023. Data were collected on demographic characteristics, risk factors, clinical examinations at admission and discharge, the complete blood count (CBC), and biochemistry. Clinical scoring was performed using the National Institutes of Health Stroke Scale (NIHSS) and Modified Rankin Scale (mRS) at both admission and discharge.

The National Institutes of Health Stroke Scale (NIHSS) is a standardized method employed to assess the severity of a stroke. It evaluates eleven distinct factors, including alertness, facial and limb movement impairments, speech difficulties, visual disturbances, and sensory sensations. The scores span from 0 to 42, indicating the following severity levels: 0: Absence of stroke symptoms;1-4: Mild stroke; 5-15: Moderate stroke;16-20: Moderate to severe stroke and 21-42: Severe stroke.

The modified Rankin Scale (mRS) assesses stroke impact on daily activities and quantifies functional outcomes.

mRS scores range from 0 (no symptoms) to 6 (death): 0: No symptoms; 1: Minimal impairments (can perform all activities); 2: Mild difficulties; 3: Moderate challenges (requires assistance); 4: Severe difficulties (inability to ambulate or manage daily tasks independently); 5: Extreme difficulties (inability to ambulate and requires constant care) and 6: Death. In addition, a new categorical variable representing stroke outcome was created based on the change in modified Rankin Scale (mRS) scores between admission and discharge. Patients were categorized as follows: (a) Improved: mRS at discharge < mRS at admission; (b) Not Changed: mRS at discharge = mRS at admission; and (c) Worsened: mRS at discharge > mRS at admission. This three-level outcome variable was included in the multivariable regression models to evaluate associations with RPR and RDW% levels.

Our observational cohort study initially included more than 153 patients with ischemic stroke. However, 35 patients were excluded for the following reasons: computed tomography (CT) artifacts and missing axial plane (29 patients), absence of CT scans (1 patient), and alternative diagnoses confirmed through MRI (3 patients, later diagnosed with a brain tumor) and the results of the CBC were unavailable for two patients. Ultimately, the final analysis was conducted on 118 patients.

Hypertension was diagnosed in patients who met at least one of the following criteria: Ongoing treatment with antihypertensive medication; Systolic blood pressure ≥ 140 mm Hg or diastolic blood pressure ≥ 90 mm Hg, confirmed on two separate measurements 24 hours after an ischemic stroke; Current treatment with insulin or oral hypoglycemic agents; Fasting blood glucose ≥ 7.0 mmol/L (126 mg/dL) on at least two separate occasions.

Hypercholesterolemia was defined as a serum cholesterol level of ≥6.2 mmol/L or higher, or the current use of cholesterol-lowering medication.

Smoking status was categorized as either current smoker or non-smoker at the time of assessment.

Ischemic heart disease was defined by a history of myocardial infarction (MI), stroke, percutaneous coronary intervention (PCI), or coronary artery bypass grafting (CABG).

Atrial fibrillation was diagnosed if there was a documented history of the condition or if it was identified on electrocardiography.

Interobserver agreement was assessed using the correlation coefficient. The NIHSS and mRS scores were evaluated at both admission and discharge. Additionally, we examined various cerebrovascular risk factors, including sex, age, hypertension, pulse pressure, diabetes mellitus, previous vascular diseases, renal failure, and heart failure.

### Calcification and inflammatory markers

**Figure 1:**
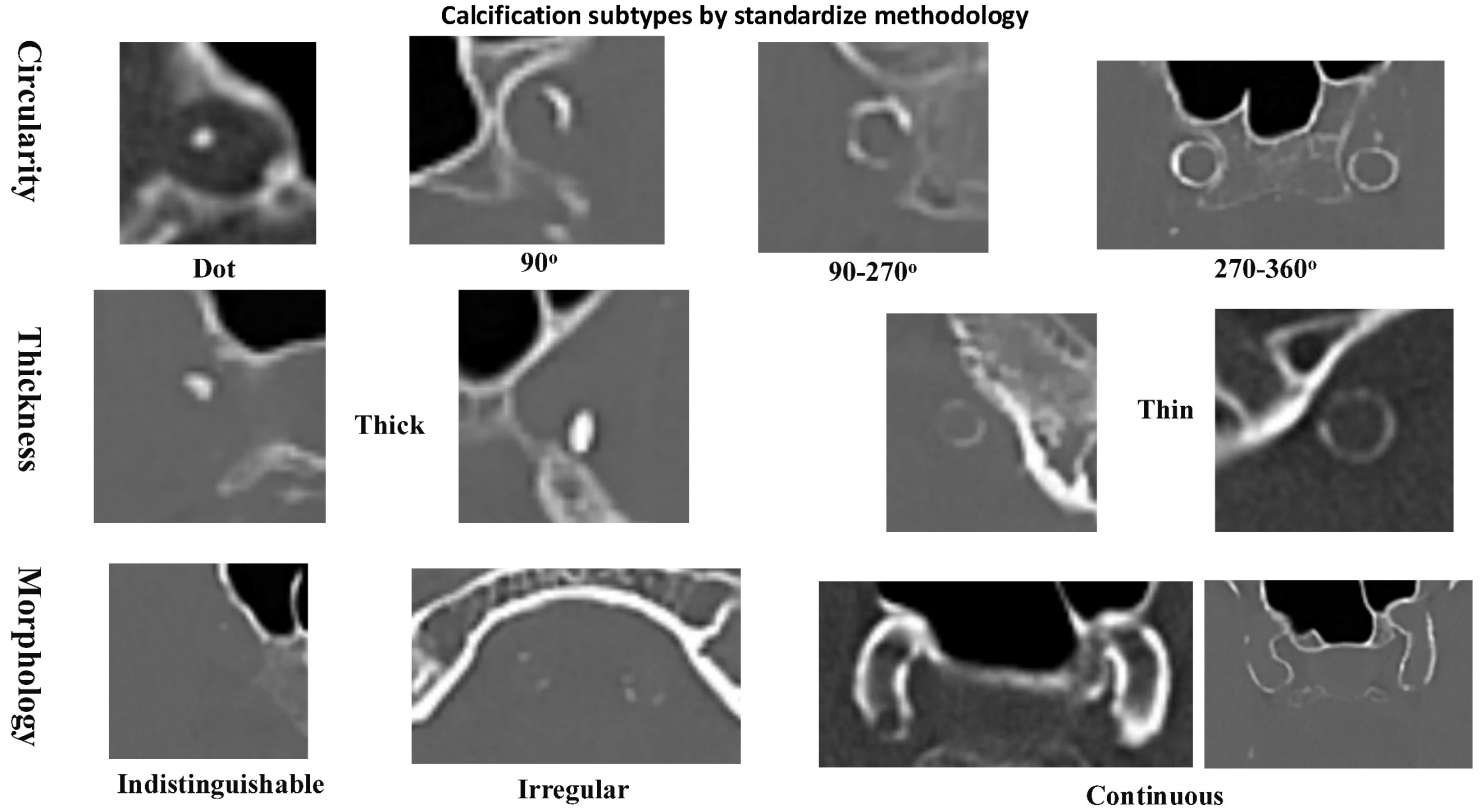
Methodology for distinguishing between calcification types in vascular structures.

The relationships between RDW% and RPR with the types of intracranial artery calcification were evaluated, as well as their impact on functional outcomes. Non-enhanced CT scans were evaluated for intracranial arterial calcification using a standardized methodology, showed in **Figure 1** (10). The interobserver variability for intracranial calcification types measurements, expressed by the intra-class correlation coefficient, was 0.967 (96% confidence interval: 0.890-0.967), with a p-value of <0.001. Based on the scoring system, arteries scoring between 1 and 6 points were classified as having mainly intimal calcification, while those scoring 7 points or higher were categorized as exhibiting mainly medial calcification. Arteries with a score of 0 were classified as lack of calcification.

Non-contrast CT scans were acquired at 120 kVp with a 300-375 mAs exposure per rotation. The slice thickness was set to 1 mm. The intracranial artery calcification patterns were categorized into intimal, medial, or absent calcification based on a previously established scoring model (10). This model evaluated the circularity of the calcification pattern: a dot was scored as 1, a 90-degree angle as 2, a 90-270-degree angle as 3, and a 270-360-degree angle as 4. The thickness of the calcification was also assessed: a thick intracranial artery greater than or equal to 1.5 mm was scored as 1, and a thin intracranial artery less than 1.5 mm was scored as 3. Additionally, the morphology of the calcification along the long axis of the artery was evaluated: an indistinguishable calcification was scored as 0, an irregular or patchy calcification was scored as 1, and a continuous calcification was scored as 4.

Informed consent was obtained from the participants or their legal representatives. RPR was calculated as the ratio of RDW% to platelet count, then multiplied by 100.

### Statistical analysis

Categorical variables were presented as numbers and percentages, while continuous variables were presented as means ± standard deviations (SD). As RPR and RDW% data showed normal distribution, comparisons between two groups were conducted using the Student's t-test. The significance of the difference between variables with more than two groups was tested using one-way ANOVA with post-hoc Bonferroni test with statistical significance indicated by a p value < 0.05. To assess the association between RPR and RDW% levels with intracranial artery calcification types and stroke outcomes, multinomial logistic regression analysis was performed, adjusting for relevant risk factors. Additionally, binary logistic regression was used to evaluate the association of RPR and RDW% with dichotomous outcome variables.

## Results

There were 118 patients with a mean age of 67.3±12.5 years, of whom 49% were female. The mRS score at admission was most frequently 2-4 (44.9%), while at discharge, it was primarily 0-2 (38.1%).

Intracranial artery calcification was mainly intimal in 15.3% (n = 18) of cases, mainly medial in 32.2% (n = 38), and mixed in 50.8% (n = 60). Only two patients with ischemic stroke were without any type of calcification on head CT. The mean RPR was 6.79 ± 2.07, while the mean RDW% was 14.64 ± 1.65, as showed in **Table 1**. Mean values of NIHSS at admission and discharge were 12.39±6.62 and 7.6±4.57, respectively.

Patients with mainly intimal calcifications had higher RPR values (8.48±2.21) compared to those with mainly medial (6.56±1.83) or mixed calcifications (6.43±1.95), p < 0.002. Additionally, patients with mainly intimal and mixed-type calcifications had higher RDW% values (14.92±1.33 and 14.79±1.72) compared to those with mainly medial calcifications (13.75±1.51), p=0.013. RPR was significantly different between intracranial artery calcification groups in the one-way ANOVA, F (3, 29) = 5.4, p = 0.002. In a multinomial logistic regression analysis adjusted for age, sex, diabetes, dyslipidemia, hypertension, smoking status, NIHSS at admission, and mRS at discharge, RPR was significantly associated with increased odds of having intimal rather than medial calcification (OR = 1.53; 95% CI: 1.06-2.11; p = 0.022), as well as intimal compared to mixed-type calcification (OR = 1.34; 95% CI: 1.03-1.75; p = 0.027).

**Figure 2:**
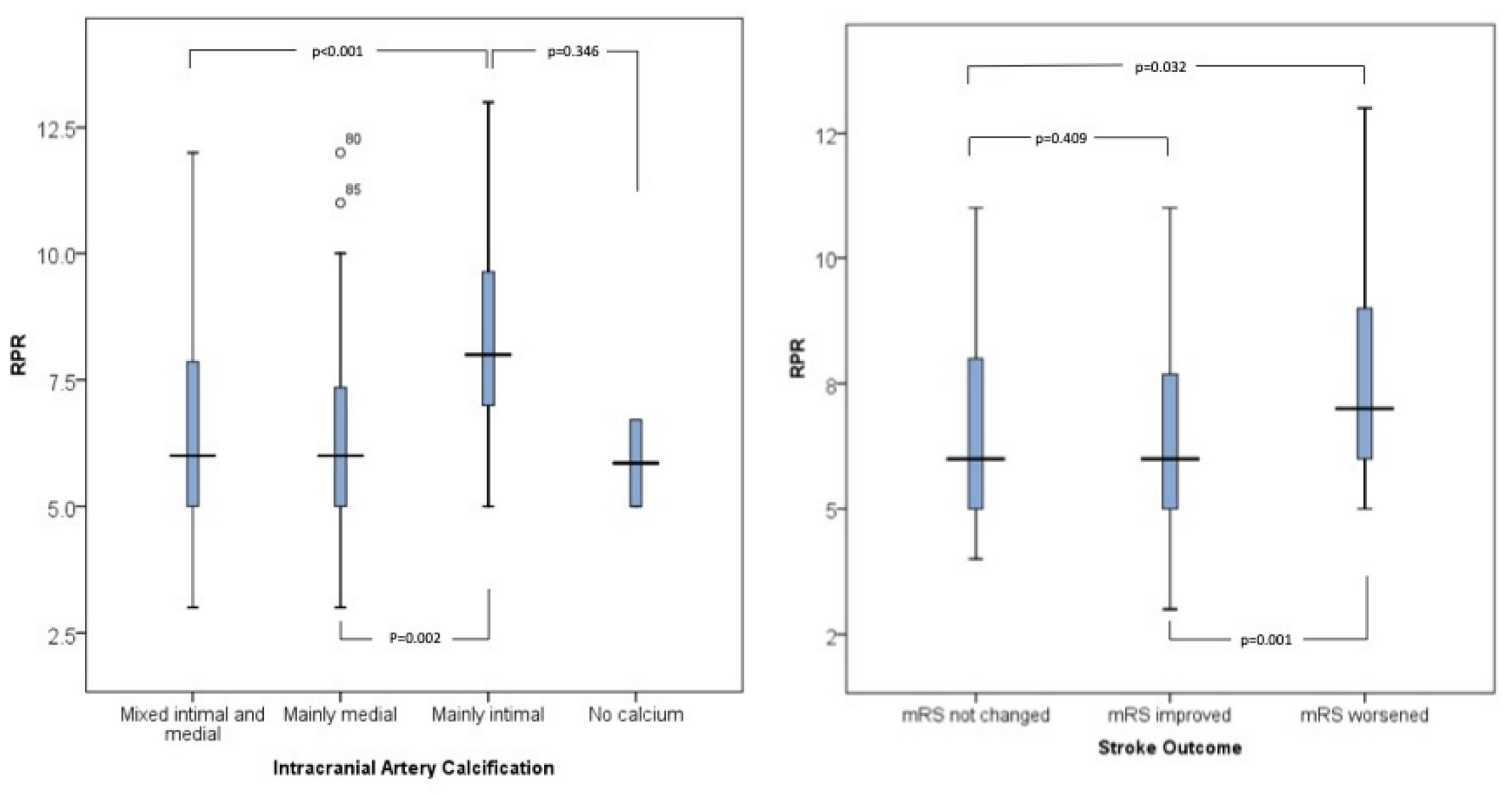
Boxpolot comparison of RPR levels across intracranial artery calcification types (left) and stroke outcome categories (right). Bars represent median and interquartile range. Statistical analysis was performed using one-way ANOVA with Bonferroni correction.

Higher values of RPR were associated with worse stroke symptoms at discharge, as measured by mRS. The mean RPR was 6.31±1.71 in patients with an mRS score of 0-2, 6.76±2.20 in those with an mRS score of 3-4, and 7.58±2.19 in those with an mRS score of 5-6 (p=0.038), (**Table 2** and **Figure 2**). When evaluating stroke outcome as a three-level variable (Improved, Not Changed, Worsened), higher RPR levels were significantly associated with a worse outcome. In the multinomial logistic regression analysis adjusted for age, sex, diabetes, dyslipidemia, hypertension, smoking, and NIHSS at admission, increased RPR was associated with a higher risk of being in the "Worsened" group compared to the "Improved" group (OR = 1.43; 95% CI: 1.09-1.88; p = 0.009) (**Table 3** and **Figure 2**).

RDW% was significantly different between intracranial artery calcification groups in the one-way ANOVA (F (3, 83.8) = 3.70, p = 0.013). Post hoc analysis with Bonferroni correction revealed significant pairwise differences; however, these associations did not remain statistically significant in the multinomial logistic regression analysis after adjusting for confounding variables (**Table 3**). Older patients and those with dyslipidemia had significantly higher values of RDW% (**Table 2**).

## Discussion

The main finding of this study is that RPR values in ischemic stroke patients were associated with mainly intimal artery calcification and, compared to mainly medial calcification and mixed-type calcification (intimal and medial). Moreover, higher RPR levels were independently associated with worse functional outcomes after stroke.

RPR, which incorporates both erythrocyte size heterogeneity and platelet count, has the potential to capture a broader range of disease-related changes, especially in situations involving inflammation and organ dysfunction 11,12.

This study might contribute to our understanding of the association between inflammatory biomarkers and intimal intracranial artery calcification. Inflammation can also have a direct effect on the stability of atherosclerotic plaque, which may lead to plaque rupture and activate in situ thrombogenesis, as well as distal artery-to-artery microembolization 13. Intimal calcification has shown to correlate with vulnerable atherosclerotic plaque phenotypes, characterized by the deposition of lipids and the infiltration of inflammatory cells 14.

**Figure 3:**
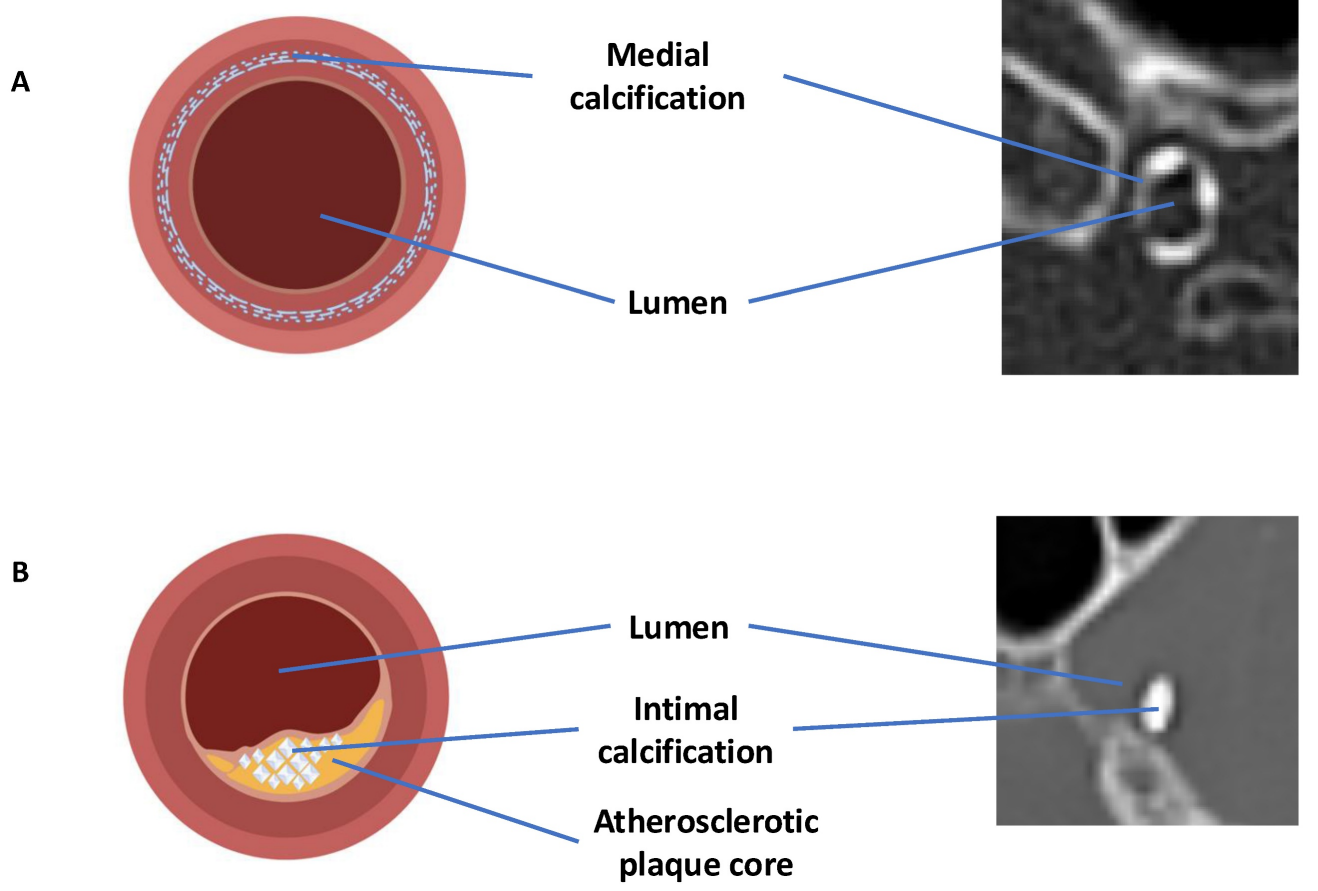
A) Calcification primarily in the medial layer; B) Calcification primarily in the intimal layer. Illustrations and CT scan examples provided. Created with BioRender.com.

Intracranial artery calcifications type can be differentiated by specific characteristics identifiable on head CT scan, by standardize methodology 10, as illustrated in **Figure 1** and **Figure 3**. In other hand, the association of RDW% and RPR with inflammatory conditions is well-established. Both intracranial artery calcification and inflammation effect the occurrence, severity and outcomes of ischemic stroke 13.

Higher levels of RPR and RDW% reflects a higher level of inflammation and larger red blood cells with less flexibility which may predispose to hypercoagulable state 7. Decades of research highlighting inflammation as a common mechanism underlying various risk factors, including dyslipidemia, have significantly advanced the scientific understanding and approach to the process of atherosclerosis 9.

Patients treated with anti-inflammatory drugs that reduced levels of high-sensitivity C-reactive protein (hsCRP) showed a significant reduction in endpoints such as stroke, myocardial infarction, and total mortality 9. High levels of RDW% and RPR are associated with elevated levels of C-reactive protein, sedimentation rate, and interleukin-6 15. So, RPR and RDW% can be used as biomarkers for the inflammatory response and the severity of inflammation 16. Several studies have shown that patients treated with thrombolysis and thrombectomy with ischemic stroke who have higher RPR and RDW% levels tend to have a worse prognosis compared to those with lower RPR and RDW% levels 17, 18.

The exact mechanism by which high levels of inflammatory biomarkers effect the severity of stroke is not entirely clear, but it is thought to play a role in hypercoagulability, oxidative stress and stability of atherosclerotic plaque. The deformability of erythrocytes may impact microcirculation, particularly collateral perfusion which might play an important role in the ischemic zone and influences stroke severity and outcomes 19. Also, smooth muscle cells induced by inflammation transform into macrophage-like cells, promoting inflammation and increasing plaque vulnerability 9.

To the best of our knowledge, no study to date has investigated the association between inflammatory biomarkers RPR and RDW% and the types of intracranial artery calcification in relation to the severity of ischemic stroke. A study has demonstrated an association between an inflammatory biomarker, hsCRP and intimal intracranial artery calcification, showing that higher levels of hsCRP correlate more strongly with mainly intimal calcification compared to medial calcification 20. Although inflammatory processes and biomarkers, such as RPR and RDW%, are hypothesized to contribute to intimal artery calcification, empirical evidence supporting this association remains limited.

**Figure 4:**
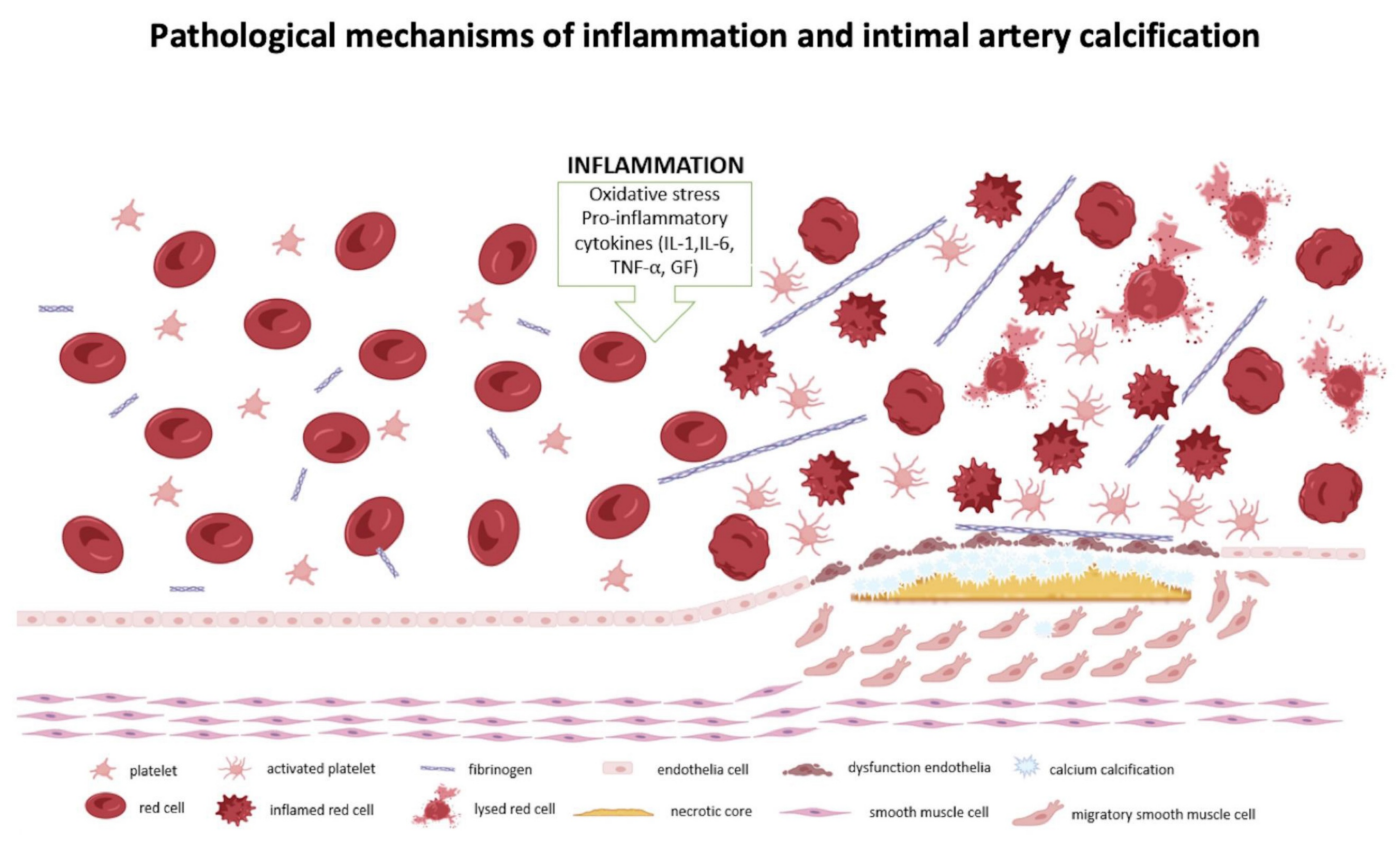
Pathological mechanisms of interrelation between inflammatory markers, changes in red blood cells and atherosclerosis. Created with BioRender.com.

In our study presence of the mainly intimal intracranial arterial calcification was associated with increased levels of inflammatory markers, compared to the mainly medial or mixed-type calcification (intimal and medial). The lack of a significant difference between the intimal and no calcification groups may be explained by the small number of patients without intracranial artery calcification in our cohort. Intimal artery calcification is associated with atherosclerosis, contributes to vessel stenosis and endothelial dysfunction, potentially leading to rupture and occlusion of the vascular lumen 16. This process triggers platelet aggregation and thrombosis, with platelets releasing inflammatory markers, such as CD40 ligands and β-globulin, which stimulate additional cytokine production and further elevate inflammation markers. Platelets, also contain a considerable amount of C-C Motif Chemokine Ligand 5 (CCL5), which, when activated, promotes monocyte adhesion and the recruitment of other immune cells 13 and as result inflammation is increased, along with blood biomarkers such as RDW% and RPR 21-23. This creates a vicious cycle that exacerbates the severity of ischemic stroke, as illustrated in **Figure 4**.

The RPR has been shown to be a potential independent predictor of 90-day outcomes in patients with ischemic stroke who have undergone thrombectomy, and independent risk factor before and after thrombolysis 24. Thus, the biomarker RPR is more comprehensive as it integrates coagulation, inflammation, oxidative stress, and other thrombotic processes, providing a broader perspective on the severity of stroke and functional outcomes 25,26.

Given the evident impact of inflammatory biomarkers (RDW% and RPR) and the role of intracranial artery calcification (intimal and medial) types in ischemic stroke, their interrelationship may have direct implications for prevention strategies, treatment approaches, and the prognosis of ischemic stroke patients.

## Conclusion

Our study showed that higher RPR are not only associated with worse outcome after ischemic stroke but also significantly correlated with intimal calcification in intracranial arteries. Since RPR and RDW% can be easily obtained from routine CBC analysis, these biomarkers have the potential to serve as accessible tools for risk stratification in ischemic stroke patients. Further validation of these findings in a multicenter study with a larger cohort would enhance their clinical applicability. Additionally, the observed relationship between RPR, RDW%, and intimal calcification might indicate a vulnerable state of intracranial artery disease, warranting further investigation into their potential role in early detection and prevention.

### Statement of ethics

This study was approved from the Ethics Committee of the Kosovo Doctors' Chamber (Protocol No. 169/2022), and informed written consent was obtained from all participating patients.

## Figures and Tables

**Table 1:** Patients clinical and demographical data.

Variables	n = 118
Age, mean±SD	67.3±12.5
Sex, n (%)	
male	50.84
female	49.16
Hypertension, n (%)	105 (89.0)
Diabetes, n (%)	46 (39)
Dyslipideami, n (%)	36 (34.6)
Smoking, n (%)	46 (39)
Atrial fibrillation, n (%)	11 (9.3)
Prior ischemic stroke, n (%)	7 (5.9)
mRS at admission, n (%)	
mRS 0 to 2	24 (20.3)
mRS 3 and 4	53 (44.9)
mRS 5 and 6	35 (29.6)
mRS at discharge, n (%)	
mRS 0 to 2	45 (38.1)
mRS 3 and 4	43 (36.4)
mRS 5 and 6	30 (25.4)
Stroke outcome, n (%)	
same	32 (27.1)
better	55 (46.6)
worse	31 (26.3)
Intracranial artery calcification, n (%)	
No calcium	2 (1.7)
Mainly intimal	18 (15.3)
Mainly medial	38 (32.2)
Mixed medial and intimal	60 (50.8)
NIHSS at admission, mean±SD	12.39±6.62
NIHSS at admission, mean±SD	7.6±4.57
RPR, mean±SD	6.79±2.07
RDW%, mean±SD	14.64±1.65

**Table 2:** Comparison of RPR and RDW% values with different clinical and radiological variables.

Variables	RPR	p	RDW	p
Age				
<65 years	6.57±1.82	0.456	14.00±1.57	**0.022**
>65 years	6.89±2.11		14.73±1.64	
Sex				
female	7.06±2.23	0.161	14.60±1.66	0.392
male	6.51±1.66		14.33±1.64	
Hypertension				
yes	6.88±2.08	0.372	14.45±1.72	0.868
no	6.30±1.93		14.52±1.01	
Diabetes mellitus				
yes	6.74±1.94	0.838	14.44±1.61	0.898
no	6.82±1.16		14.48±1.68	
Dyslipidemia				
yes	7.21±2.36	0.077	14.95±1.89	**0.014**
no	6.44±1.82		14.09±1.47	
Smoking				
yes	6.56±1.76	0.331	14.41±1.44	0.803
no	6.95±2.26		14.49±1.80	
mRS at admission				
0 to 2	6.18±1.56	0.204	14.42±1.52	0.853
3 to 4	6.75±2.43		14.48±1.48	
5 to 6	7.19±2.43		14.29±1.77	
mRS at discharge				
0 to 2	6.31±1.71	**0.038**	14.22±1.51	0.273
3 to 4	6.76±2.20		14.80±1.83	
5 to 6	7.58±2.19		14.40±1.60	
Stroke outcome				
mRS not changed	6.62±1.97	**0.003**	14.85±1.84	0.345
mRS improved	6.22±1.74		14.26±1.57	
mRS worsened	7.84±2.34		14.43±1.57	
Calcification type				
no calcium	5.85±1.20	**0.002**	14.50±0.28	**0.013**
mainly intimal	8.48±2.21		14.92±1.33	
mainly medial	6.56±1.83		13.75±1.51	
mixed medial and intimal	6.43±1.95		14.79±1.72	

**Table 3:** Regression analysis for the association of RPR and RDW% with vascular risk factors, intracranial calcification types, and stroke outcome. All models were adjusted for age, sex, smoking, diabetes, hypertension, hypercholesterolemia, atrial fibrillation, NIHSS score at admission, and mRS at admission. Analysis of the data with more than two groups was performed by multinomial regression analysis. All the other analyses were performed by binary logistic regression.

Variables	RPR (adjusted)		p	RDW (adjusted)		p
	OR	(95% CI)		OR	(95% CI)	
Age (>65 years old)	1.08	(0.88-1.31)	0.452	**1.36**	**(1.04-1.77)**	**0.024**
Sex (male)	0.89	(0.73-1.10)	0.304	0.9	(0.70-1.15)	0.406
Smoking	1.38	(0.36-5.32	0.632	1.18	(0.24-5.80)	0.835
Diabetes	0.93	(0.75-1.16)	0.537	0.71	(0.95-1.24)	0.537
Hypertension	1.16	(0.84-1.60)	0.353	9.7	(0.64-1.46)	0.474
Hypercholesterolemia	1.2	(0.97-1.47)	0.081	**1.4**	**1.06-1.86)**	**0.018**
Prior vascular disease	1.14	(0.91-1.44)	0.243	0.76	(0.55-1.05)	0.106
Atrial fibrillation	1.54	(0.16-14.15)	0.701	0.61	(0.89-4.16)	0.612
Calcification type*						
mainly intimal vs. mainly medial	**1.53**	**(1.06-2.11)**	**0.022**	0.824	(0.54-1.24)	0.361
mainly intimal vs. mixed	**1.34**	**(1.03-1.75)**	**0.027**	0.84	(0.37-1.90)	0.681
mainly intimal vs. no calcification	1.23	(0.52-1.84)	0.354	0.91	(0.76-1.15)	0.406
Stroke outcome*						
mRS worsende vs. mRS improved	**1.43**	**(1.09-1.88)**	**0.009**	1.12	(0.83-1.51)	0.427
mRS worsende vs. mRS not changed	**1.34**	**(1.03-1.75)**	**0.027**	0.99	(0.98-1.00)	0.227
